# *Enterococcus faecalis*–associated infective aortic endocarditis as a cause of arterial thromboembolism in a cat

**DOI:** 10.1007/s11259-026-11208-1

**Published:** 2026-04-13

**Authors:** Didem Algan, Tuğba Varlik, Mehmet Emin Akkaş, Juon Abbass, Melek Zübeyde Demirci, Kamil Tayfun Carli, Zeki Yilmaz

**Affiliations:** 1 Department of Internal Medicine, Faculty of Veterinary Medicine, Bursa Uludag University, Bursa, 16059 Turkey; 2 Department of Microbiology, Faculty of Veterinary Medicine, Bursa Uludag University, Bursa, Türkiye Turkey

**Keywords:** *Enterococcus faecalis*, Infective aortic valve endocarditis, Arterial thromboembolism, Cat

## Abstract

Infective endocarditis (IE) is a rare but potentially fatal cause of feline arterial thromboembolism (ATE), most commonly affecting the mitral or aortic valves. Although *Enterococcus faecalis* is rarely reported in cats, it can induce severe endocardial infection and systemic embolic complications. This case report describes a one-year-old neutered male British Longhair cat that presented with acute hindlimb paralysis and dyspnoea lasting one day. The cat had a three-month history of lower urinary tract infection, but no known cardiac disease. On admission, all the classical signs of arterial obstruction were evident. Laboratory tests revealed neutrophilic leukocytosis and markedly increased serum amyloid A, indicating systemic inflammation. Echocardiography revealed thickened aortic valve leaflets with irregular vegetations and severe regurgitation, with no evidence of intracardiac thrombus. Left ventricular systolic function assessed by two-dimensional speckle-tracking echocardiography was mildly reduced compared with reference values. Blood culture confirmed the presence of *E. faecalis*, which was susceptible to β-lactams but intrinsically resistant to cephalosporins. Initial empirical therapy included furosemide, low-molecular-weight heparin, and broad-spectrum antibiotics. This was later adjusted to ampicillin–sulbactam, and metronidazole upon receiving the culture results. This report emphasises the importance of considering xone,*E. faecalis*-associated infective aortic endocarditis in the diagnostic and therapeutic evaluation of feline ATE cases.

## Background

Arterial thromboembolism (ATE) is a life-threatening vascular event in both humans and animals. In cats, it most often follows hypertrophic cardiomyopathy (HCM), the most common feline cardiomyopathy, and typically manifests as acute hindlimb paralysis, pain, and loss of femoral pulses (Fuentes 2012). Although cardiogenic ATE is most frequent, septic thromboembolism secondary to infective endocarditis (IE) should also be considered.

IE is a rare but severe disease in cats, often underdiagnosed due to nonspecific clinical signs (Fuentes 2012). In human medicine, the epidemiology of IE has changed in recent decades: while *Staphylococcus aureus* remains dominant, *Enterococcus faecalis* (*E. faecalis*) now represents the third leading cause worldwide, accounting for 10–15% of cases (Herrera-Hidalgo et al. 2023). *E. faecalis*, a Gram-positive, facultative anaerobe of intestinal flora (Arias and Murray 2012), is responsible for about 90% of enterococcal IE and frequently associated(Fuentes 2012) with embolic complications (Holland et al. 2016; Dahl et al. 2019).

In veterinary medicine, IE prevalence is extremely low—0.006–0.018% in cats and 0.05–6.6% in dogs (Palerme et al. 2016; van Loon et al. 2020). Reported pathogens include *Bartonella spp.*, *Staphylococcus spp.*, *Streptococcus spp.*, and *Escherichia coli* (Sereda et al. 2022; Colella et al. 2023). *Enterococcus* infections are rarely described in cats (Sereda et al. 2022; Colella et al. 2023; van Loon et al. 2020) or dogs (Tessier-Vetzel et al. 2003), mostly affecting the mitral valve. No previous reports have linked *E. faecalis* to aortic-valve IE with ATE in cats. This report describes a young cat with bilateral hindlimb ATE secondary to *E. faecalis* aortic valve endocarditis, emphasizing this organism as an under-recognized feline pathogen.

## Case history

A 1-year-4-month-old neutered male British Longhair cat (4.1 kg) was admitted with acute hindlimb paralysis and dyspnea of one-day duration. The cat had a three-month history of lower urinary tract infection (LUTI) but no known cardiac disease.

**Fig. 1:**
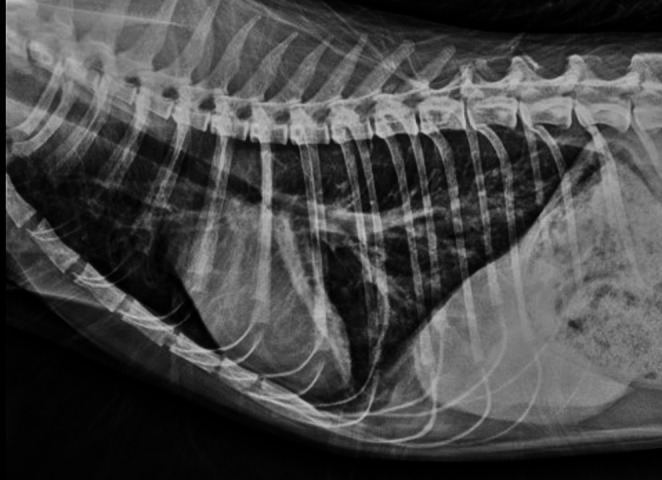
Lateral thoracic radiographs obtained at presentation, showing normal cardiac size, mild dilation of the ascending aorta, and an interstitial-bronchial pulmonary pattern

On presentation, body temperature was normal with moderate dehydration and no palpable hindlimb pulses. The five classics “P’s” of arterial obstruction—pain, paralysis, pallor, pulselessness, and poikilothermia—were evident (Fuentes 2012). CBC revealed neutrophilic leukocytosis (Table 1) and markedly increased serum amyloid A (fSAA), indicating systemic inflammation. Biochemistry showed hyperproteinemia, stress hyperglycemia, and mild hepatic-renal alterations; cardiac troponin I and Pro-BNP were within reference ranges (Table 1). FIV, FeLV, and FCoV tests were negative. Thoracic radiographs showed an interstitial bronchial pattern observed in the lung fields without cardiomegaly. The ascending aorta appeared mildly dilated, mimicking post-stenotic dilatation (Fig. 1).

**Table 1:** Selected clinical, hematological, and serum biochemical findings in a cat with infective endocarditis (IE)

Parameters	Cat with IE	References
Vital signs*		
Temperature C	38.0	38.1–39.2
Heart rate bpm	217	< 220
Respiration rpm	75	< 40
CBC results **		
WBC x10^3^/uL	25.12	5.5–19.5
Lymphocyte	0.401	1.5–7.0.5.0
Monocyte	0.326	0–0.85.85
Neutrophil	24.17	2.5–12.5
Eosinophil	0.15	0–1.5.5
Basophil	0.06	0–0.2.2
RBC x10^6^/uL	9.11	5.0–10.0
Hgb gr/dL	12.1	8.0–15.0
Hct %	36.6	24.0–45.0
MCV fL	40.2	39.0–55.0
MCHC gr/dL	33.0	30.0–36.0
RDW_SD fL	20.4	29–35
Platelet x10^3^/uL	56.0	200–800
MPV fL	10.4	8.2–16.3
PCT %	0.05	0.0–0.79.0.79
Serum biochemistry*		
Total protein g/dL	9.8	6.0–7.9
ALP IU	37	9–53
ALT IU	355	22–84
Glu mg/dL	189	60–120
Cr mg/dL	2.0	0.8–1.8
BUN mg/dL	49.6	17.6–32.8
fSAA ug/mL	121.8	< 5.0
cTnI ug/dL	0.016	0.00–0.16
ProBNP pmol/L	112	< 50

*CBC *complete blood count, *WBC* white blood cel*, RBC *red blood cell,* HGB *hemoglobin*, HCT *hematocrit,* MCV *mean corpuscular volume,* MCHC *mean corpuscular hemoglobin concentration*, RDW-SD *red cell distribution width–standard deviation,* PLT *platelet*, MPV *mean platelet volume*, PCT *plateletcrit,* TP *total protein*, ALP *alkaline phosphatase*, ALT *alanine aminotransferase*, GLU *glucose*, CR *creatinine*, BUN *blood urea nitrogen*, fSAA *feline serum amyloid A*, cTnI *cardiac troponin I*, ProBNP *n-terminal pro–b-type natriuretic peptide. * (Fielder 2024) **(Weiss and Wardrop 2010)

**Fig. 2:**
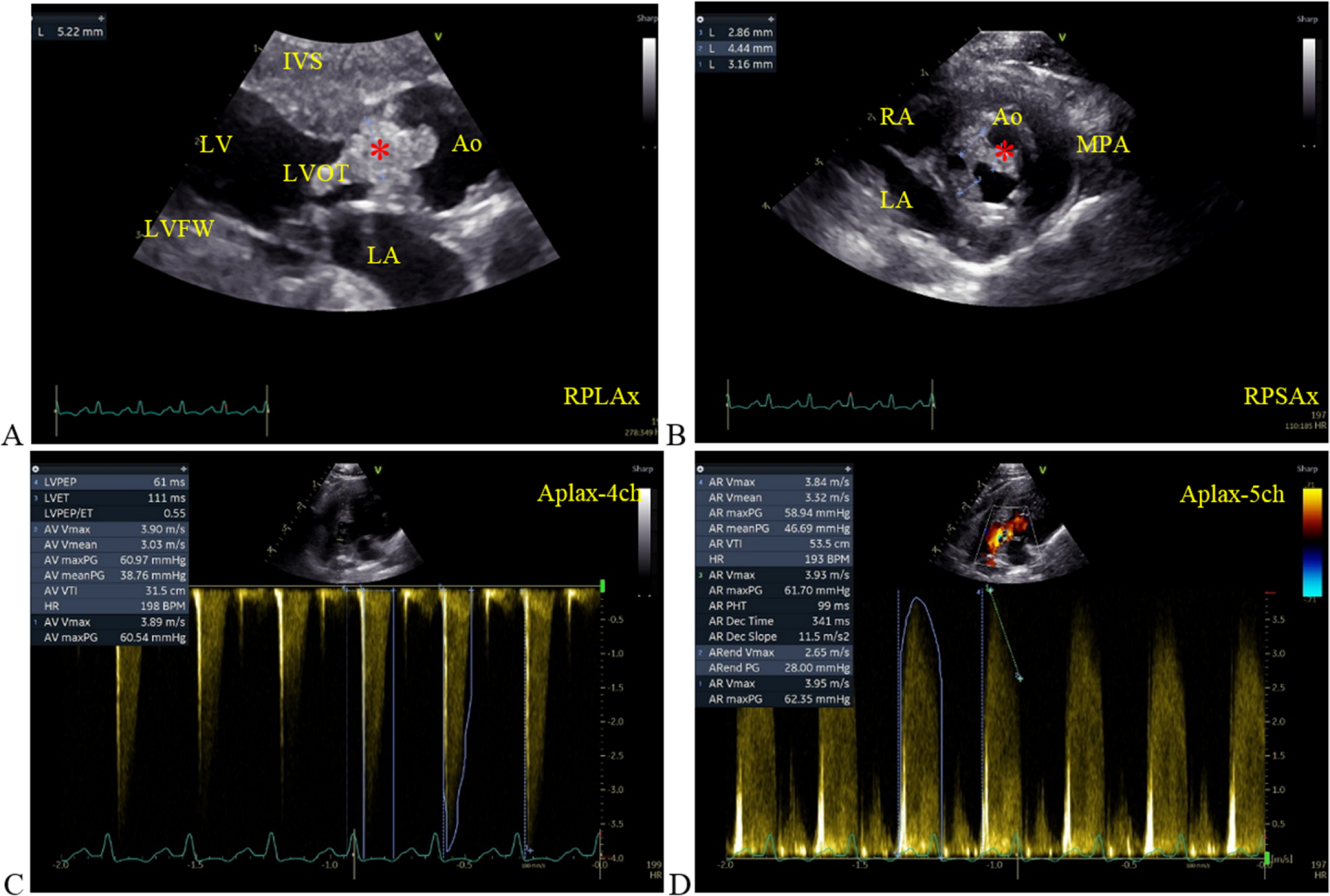
(**A**) Right parasternal long-axis five-chamber view (RPLAx) showing aortic valve (Ao, *) vegetation, thickening (5.22 mm), and deformation, which narrows the left ventricular outflow tract (LVOT), affects aortic inflow, and has resulted in post-stenotic aortic dilation. (**B**) Right parasternal short-axis (RPSAx) view at the level of the aorta (*), showing vegetative lesions and degeneration involving all three aortic cusps (non-coronary and left and right coronary cusps). (**C**) Apical five-chamber view (Aplax) with pulsed-wave Doppler across the aortic valve, demonstrating increased peak velocity and pressure gradient, consistent with dynamic obstruction. (**D**) In the same Aplax view, color Doppler demonstrates marked turbulent flow across the aortic valve and vegetation area, while continuous-wave Doppler recording of the aortic flow confirms the presence of severe aortic valvular regurgitation

 ECG showed sinus rhythm. Echocardiography was performed without sedation (Vivid S60N Ultra Edition, 12 S cardiac probe, GE Healthcare, Norway; Penta Electronic, distributor in Istanbul/Türkiye). All images and video records were stored and transferred to the computer with an analysis software (EchoPac™ PC, GE HealthCare, version 204), to offline measurements, as reported in the previous studies (Bach et al. 2021; Yilmaz et al. 2025). These measurements demonstrated normal cardiac chamber dimensions, mild LV free wall (LVFW) thickening (Table 2), and no intracardiac thrombus. Two-dimensional and Doppler studies revealed thickened aortic valve leaflets with irregular vegetations (Fig. 2 A, B) and increased inflow velocity (3.9 m/s; pressure gradient 60.9 mmHg; Fig. 2 C) with severe regurgitant flow (3.8 m/s, gradient 58.9 mmHg; Fig. 2D), indicative of aortic valve endocarditis.

**Table 2:** Selected echocardiographic measurements in a cat with arterial thromboembolism due to infective endocarditis

Parameters	Cat	References *
Body weight Kg	4.1	4.1 ± 1.1
IVSd (mm)	4.6	4.6 ± 0.6
LVIDd (mm)	13.1	15.9 ± 2.3
LVPWd (mm)	6.8	4.3 ± 0.7
IVSs (mm)	5.7	7.4 ± 1.3
LVIDs (mm)	6.9	8.1 ± 1.8
LVPWs (mm)	9.6	7.5 ± 1.1
LA (mm)	11	12 ± 1
Ao (mm)	11	10 ± 1
LA/Ao	1.0	0.9 ± 0.1
LA vol (mL)	1.0	1.0
EF % (Teich)	83	70.9-87.2
FS%	47	49±7
MPA Vmax m/s	1.5	0.5-1.6
AV Vmax m/s	3.9	1.1 ± 0.2
MV E/A	1.0	1.5 ± 0.3
PV S/D ratio	1.1	0.90 ± 0.29
TDI MV annulus septal – E/E’ Avg	14.3	<15
TTSep mm	4.3	3.3 – 6.4**
TTLat mm	1.7	3.2 – 7.7**

*IVSD* interventricular septum diastole, *LVIDd *left ventricular internal diameter diastole, *IVSs *interventricular septum systole, *LVIDs *left ventricular internal diameter systole, *LVPWd *left ventricular post wall diastole, *LVPWs *left ventricular post wall systole,*FS*fractional shortening, *LA*left atrium, *Ao*Aor,* MPA *main pulmoner artery, *LA/Ao *Left atrium/ aorta ratio, *LA vol *left atrial volume, *EF* ejection fraciton, *EDV *end-diastolic volume, *ESV *end-systolic volume, *AoVmax *Aort maximal velocity, *Mitral E/A *mitral early ventricular filling (E) and late atrial contraction (A), *LA vol *left atrial volume, *LAA Vmax *left atrial appendage maximal flowvelocity, *TT*_*Sep*_tissue tracking of basal interventricular septum,* TT*_*Lat*_tissue tracking of the basal lateral segment of the left ventricular free wall

*Boon, 2011; ** Bach et al. 2021

**Fig. 3:**
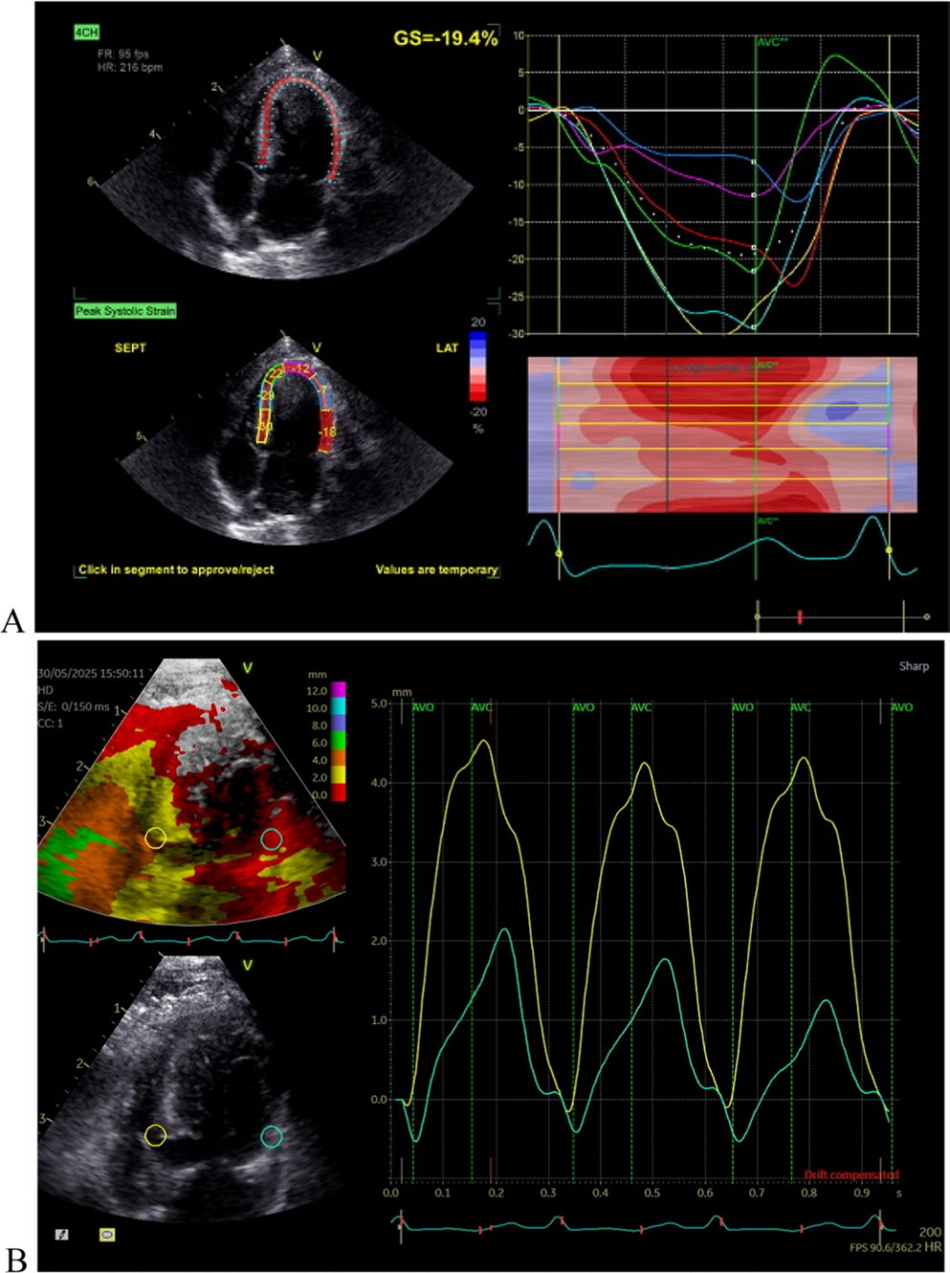
(**A**) Left ventricular (LV) longitudinal strain profiles in six segments of the four-chamber view obtained by two-dimensional speckle tracking echocardiography show a slightly reduced global longitudinal strain (GLS: −19.4%; reference value < − 21%). While the septal segments display normal strain values, the strain in the LV free wall (LVFW) segments (basal, mid, and apical lateral) is comparatively reduced. In the color-coded strain map, red areas indicate well-contracting myocardial regions, whereas blue and light blue areas represent segments with reduced contractility. Additionally, the basal segment (line with a dark red and dark blue in the strain curve) of the LVFW exhibited post-systolic shortening (PSS; yellow arrow), as indicated by a negative strain pattern occurring after aortic valve closure (AVC). (**B**) Tissue tracking (TT) imaging obtained from an apical four-chamber view with target points placed at the basal septum (TT_Sep_) and LVFW (TT_Lat_). The yellow circle indicates normal contraction of the basal septal segment, whereas the green circle demonstrates reduced longitudinal displacement in the LVFW segment, consistent with the GLS findings

 Left ventricular (LV) systolic function appeared preserved according to conventional echocardiographic parameters, including ejection fraction (EF%) and fractional shortening (FS%). However, the two-dimensional speckle-tracking echocardiography (2D-STE)–derived global longitudinal strain (GLS = –19.4%; Fig. 3) was mildly reduced. Segmental strain analysis revealed that strain values in the basal, mid, and apical segments of the LVFW were lower than those in the interventricular septal segments. In this context, more negative strain values indicate better myocardial contractility across LV segments (Fig. 3A). LV longitudinal systolic function was also assessed using the tissue tracking (TT) module, which quantifies myocardial longitudinal displacement by integrating tissue velocity along the ultrasound beam axis. Tissue velocity imaging cine loops were obtained from an LV-focused apical four-chamber view and analyzed offline. Systolic displacement values were measured at the septal (TT_sep_) and lateral (TT_lat_) mitral annular segments using ECG and Doppler-based valve timing and were depicted in millimeters by the software (Fig. 3B), as reported (Bach et al. 2021; Varlik et al. 2026). 

Renal arterial hemodynamics were evaluated using pulsed-wave Doppler ultrasonography of the interlobular arteries. Blood flow velocities were measured to calculate the resistive index (RI) and pulsatility index (PI), which reflect intrarenal vascular resistance. RI was calculated as (PSV – EDV)/PSV, and PI as (PSV – EDV)/mean velocity, where PSV and EDV represent the peak systolic and end-diastolic velocities, respectively. In this case, RI (0.99) and PI (2.75) exceeded the reported reference limits (< 0.70 and < 1.30, respectively) (Evangelista et al., 2022).

Due to suspected IE and hemato-biochemical evidence of systemic inflammation, empirical therapy was initiated immediately (Habib et al. 2015; Chae et al. 2025). Broad-spectrum antibiotics, including ceftriaxone, ampicillin, and metronidazole, were started. Supportive care included furosemide (2 mg/kg IV BID) for pulmonary edema, butorphanol (0.04 ml/kg SC BID) for analgesia and sedation, and isotonic saline for rehydration, and low-molecular-weight heparin (LMWH, 150 IU/kg, SC, q6h) to prevent further thrombosis and aid clot resolution (Fuentes 2012). Before initiating therapy, blood samples were aseptically collected from two separate venipuncture sites. Each sample was cultured independently on 5% sheep-blood agar. After 24–48 h incubation at 37 °C, small translucent colonies of Gram-positive cocci in pairs and short chains were observed, and results from both sites were concordant. Identification by the BD Phoenix™ system confirmed *E. faecalis*. Antimicrobial susceptibility testing (CLSI guidelines) showed intrinsic resistance to cephalosporins but susceptibility to selected β-lactams, including ampicillin and amoxicillin. Based on culture and susceptibility results, antibiotic therapy was revised according to culture and susceptibility results to ampicillin–sulbactam (20 mg/kg, IV, BID), and metronidazole (30 mg/kg, IV, BID) while supportive care was continued unchanged. The cat remained hospitalized for three days under intensive care. Unfortunately, due to financial constraints, the owner discontinued hospitalization, and follow-up evaluation was not possible.

## Discussion

 This case describes the first report of *E. faecalis* IE involving the aortic valve in a cat complicated by ATE. The acute onset of bilateral hindlimb paralysis and systemic inflammatory findings emphasizes that IE should be included in the differential diagnosis of feline ATE, even in the absence of HCM or other structural cardiac disease Considering that HCM is classically diagnosed based on increased thickness of the IVS, the LVFW, or both, the isolated LVFW thickening observed in this case was interpreted as pseudohypertrophy secondary to dehydration (Sugimoto et al. 2019) and/or as a response to the hemodynamic stress associated with severe aortic regurgitation. Severe aortic regurgitation typically induces eccentric LV remodeling as a consequence of chronic volume overload. However, regional variations in myocardial wall stress and intracavitary flow dynamics may result in heterogeneous myocardial adaptation, which could explain the localized thickening observed in the LVFW without a corresponding increase in IVS thickness or LV end-diastolic dimension (Marigliano et al. 2024).

Diagnosis of IE was established using the modified Duke criteria for cats (Palerme et al. 2016). Major criteria included echocardiographic evidence of vegetative and destructive valvular lesions with regurgitation, while ATE represented a minor vascular phenomenon. Absence of intracardiac thrombi and normal cardiac biomarkers supported a septic rather than cardiogenic origin. Leukocytosis, elevated fSAA, and a positive blood culture further confirmed the diagnosis. While *Bartonella*, *Streptococcus*, and *Staphylococcus* are the most frequent agents in feline IE (Sykes et al. 2006; van Loon et al. 2020; Colella et al. 2023), this case extends current knowledge by identifying *E. faecalis* as a new feline pathogen. Although the modified Duke criteria can have limited sensitivity in *E. faecalis* IE (Delgado et al. 2023), this organism remains a leading cause of enterococcal IE, accounting for approximately 90% of infections (Dahl et al. 2022). Therefore, routine blood culture screening markedly improves diagnostic yield (Dahl et al. 2019) Aortic valve involvement is frequently observed in both canine and feline IE (Sykes et al. 2006; Palerme et al. 2016; Sereda et al. 2022). The present case parallels recent feline reports with mitral–aortic vegetations and ATE (Sereda et al. 2022) but is distinguished by confirmed *E. faecalis* infection, previously described only in a cat with *E. hirae* multivalvular endocarditis (Tessier-Vetzel et al. 2003) and a dog with *E. faecalis* mitral valve IE (Tessier-Vetzel et al. 2003). Echocardiographic findings of irregular aortic vegetations, severe regurgitation, and elevated transvalvular velocity are consistent with human and veterinary descriptions (Dahl et al. 2022; Delgado et al. 2023; Chae et al. 2025).

The most probable source of bacteremia in this case was a prior LUTI, as previously reported in enterococcal endocarditis (Fernández Guerrero et al. 2007; Ceci et al. 2015). A urine culture was not performed at the time of the LUTI, and thus the specific causative isolate could not be identified. Alternative routes such as gastrointestinal or biliary translocation were considered but were not supported by the clinical and hemato-biochemical findings. Catheter-related contamination, although theoretically possible, was excluded (Milbrandt 1998). Cardiac dysfunction is recognized as a major prognostic factor in infective endocarditis (Lauridsen et al. 2018) In the present case, conventional systolic indices, including EF% and FS%, remained within the reference ranges; however, the reduced LV global (GLS) and segmental longitudinal (TT_Lat_) strains suggested early systolic impairment. This finding underscores the diagnostic value of advanced echocardiographic techniques such as 2D-STE for detecting subtle myocardial dysfunction (Lauridsen et al. 2018; Bach et al. 2021; Yilmaz et al. 2025). Because myocardial fibers are predominantly arranged in a longitudinal orientation, longitudinal deformation is particularly sensitive to early myocardial injury and may decline before changes in LV EF become apparent. Accordingly, the reduced LV-GLS observed in this case is consistent with previous reports demonstrating that GLS is a more sensitive indicator of subclinical myocardial dysfunction in pathological conditions such as aortic regurgitation, even when LV EF remains preserved (Lauridsen et al. 2018; Marigliano et al. 2024; Varlik et al. 2026). The observed reduction in myocardial strain may be related to inflammatory cytokine activity, microvascular ischemia, or increased hemodynamic stress associated with infected cardiac valves (Habib et al. 2015; Delgado et al. 2023; Marigliano et al. 2024).

In this case, the elevated RI and PI values, when interpreted alongside increased serum BUN and creatinine concentrations, were considered compatible with renal involvement. These changes may reflect elevated intrarenal vascular resistance (Evangelista et al. 2022), most likely due to systemic inflammation, as reflected by hyperproteinemia, elevated fSAA and neutrophilic leukocytosis, dehydration, and potentially reduced renal perfusion (Evangelista et al. 2022; Lauridsen et al. 2018), although other factors—such as RAAS activation, increased sympathetic tone, and renal artery stenosis or obstruction—cannot be excluded.

Antimicrobial therapy in this case was guided by blood culture results and susceptibility testing. In light of current clinical guidelines and evidence from human IE, a combination of ampicillin and sulbactam was selected to optimize bactericidal activity while minimizing aminoglycoside-associated nephrotoxicity. Ampicillin-sulbactam, alone or in combination with ceftriaxone is recommended in patients with enterococcal IE as an alternative to traditional regimens containing gentamicin, particularly in those with prolonged disease duration or higher risk for renal injury (Gavalda et al. 2007; Baddour et al. 2015; Habib et al. 2015; Mirma et al. 2022; Delgado et al. 2023). This double β-lactam regimen provides synergistic bactericidal effects through complementary saturation of penicillin-binding proteins, and has been shown to achieve effective clinical outcomes with a more favorable safety profile compared with aminoglycoside-containing combinations (Gavalda et al. 2007; Mirna et al. 2022). Owing to these considerations, and given the absence of high-level aminoglycoside resistance in our isolate, this approach to antibiotic selection was considered the most appropriate in the current case. Low-molecular-weight heparin was used for anticoagulation and monitored with thromboelastography (Eralp Inan et al. 2016; Yilmaz et al. 2017). Although pimobendan could enhance systolic performance, it was avoided due to the risk of increasing aortic flow velocity and dislodging vegetative fragments (Miller et al. 2004).

In conclusion, this case report reveals for the first time *E. faecalis* as a rare but significant cause of feline IE and ATE. The overlap with cardiogenic ATE underscores the importance of considering septic etiologies in cats presenting with acute paralysis and systemic inflammation. Early echocardiography and blood cultures are critical for full diagnosis and monitoring. Greater clinical awareness and further research are needed to improve recognition and management of enterococcal IE in veterinary medicine.

## Data Availability

The datasets and materials used and/or analyzed during the current study are available from the corresponding author upon reasonable request.
